# The Ohio State Dental Society

**Published:** 1882-01

**Authors:** 


### THE OHIO STATE DENTAL SOCIETY.

The Sixteenth Annual Meeting of this body was held Dec. 7th, 8th, and 9th; and in most, if not all respects, it was the best meeting of this Society ever held. The attendance was larger than ever before, amounting in all to about one hundred and fifty; quite a large number of new members were added; a goodly number were present as visitors, not only from Ohio, but from Indiana, Kentucky, Michigan, and Pennsylvania. A very warm and cordial sympathy, and fraternal feeling exists between the Dental Societies of these different States; and usually at the annual meeting of each of these States there will be found in attendance representatives from some, if not all, of these States. More work was accomplished than at any meeting for many years.

Quite a number of excellent papers were read; some upon the subjects of the programme, and some upon other subjects; prominent among the latter was one by Dr. Geo. Watt on the affect Ammonia as exhibited in the mouth; and one by Dr. J. A. Robinson, of Jackson, Mich.

The former was a masterly presentation of the subject, which has heretofore only been outlined by Dr. W., and not attempted by any one else. This is one of Dr. W.'s best productions, and will doubtless pass into the literature of the profession, and take a place by the side of his deliverances upon "Topical Remedies," written nearly thirty years ago, and which the discoveries and improvements since made, have not set aside nor superseded.

Dr. Robinson is one of the oldest practitioners and writers in the profession. He wields a facile pen, and in his writings presents a vivid flow of practical facts and suggestions. There were present Dr. Rawls, of Ky.; Profs. Hunt and Chappell and Dr. Harriot, of Indiana; Drs. French and King, of Pa. The presence of these gentlemen added much to the interest of the occasion.

The record of the transactions of this meeting will be promptly issued, and we advise every one, at least in our State, to procure a copy, and thus learn just what was done, at least so far as it is possible from the published reports.

